# Adsorptive Capacity, Inhibitory Activity and Processing Techniques for a Copper-MOF Based on the 3,4-Dihydroxybenzoate Ligand

**DOI:** 10.3390/molecules27228073

**Published:** 2022-11-21

**Authors:** Estitxu Echenique-Errandonea, Sara Rojas, Víctor Karim Abdelkader-Fernández, Manuel Pérez-Mendoza, Ricardo F. Mendes, Paula Barbosa, Filipe Figueiredo, Flávio Figueira, Filipe A. Almeida Paz, José Manuel Delgado-López, Antonio Rodríguez-Diéguez, José Manuel Seco

**Affiliations:** 1 Departamento de Química Aplicada, Facultad de Química, Universidad del País Vasco UPV/EHU, Paseo Manuel Lardizabal, N° 3, 20018 Donostia-San Sebastián, Spain; 2 Departamento de Química Inorgánica, Facultad de Ciencias, Universidad de Granada, Av. Fuentenueva S/N, 18071 Granada, Spain; 3 Department of Chemistry, CICECO—Aveiro Institute of Materials, University of Aveiro, 3810-193 Aveiro, Portugal; 4 Department of Materials & Ceramic Engineering, CICECO—Aveiro Institute of Materials, University of Aveiro, 3810-193 Aveiro, Portugal

**Keywords:** metal-organic frameworks, material processing, adsorption capacity, antibacterial activity

## Abstract

Due to the fast, emerging development of antibiotic-resistant bacteria, the need for novel, efficient routes to battle these pathogens is crucial; in this scenario, metal-organic frameworks (MOFs) are promising materials for combating them effectively. Herein, a novel Cu-MOF—namely **1**—that displays the formula [Cu_3_L_2_(DMF)_2_]_n_ (DMF = *N*,*N*-dimethylformamide) is described, synthesized by the combination of copper(II) and 3,4-dihydroxybenzoic acid (H_3_L)—both having well-known antibacterial properties. The resulting three-dimensional structure motivated us to study the antibacterial activity, adsorptive capacity and processability of the MOF in the form of pellets and membranes as a proof-of-concept to evaluate its future application in devices.

## 1. Introduction

Infections caused by pathogenic bacteria have been considered one of the most concerning global threats affecting public healthcare. To tackle this problem, over the years, antibiotics have been administered to treat bacterial infections. However, due to the growing resistance of pathogens to conventional antibiotics, treatments often become less effective or even ineffective. Consequently, the need to develop new effective solutions to broaden the range of antibacterial agents and combat infections more efficiently has become very important [1,2].

In this context, coordination chemistry offers a great alternative in the design of new bioactive compounds [3,4]. In particular, our research group has worked with such materials in fields as diverse as diabetes [5], cancer [6], Alzheimer’s [7], inflammatory processes [8], Chagas disease and leishmaniasis [9]. In recent years, metal-organic frameworks (MOFs) have been presented as promising materials for the treatment and detection of disease-related conditions. Bacterial activity in MOFs can be conducted by two other mechanisms [2,10]: In the most common mechanism, MOFs act as reservoirs of bioactive metal ions and their gradual degradation leads to a controlled release, resulting in a sustained antibacterial effect with high efficiency. The other mechanism supports the theory that antibacterial activity results from interactions between the bacterial surface and the active site of MOF’s surface. In this latter case, active centres present in MOFs are stabilised by strong chemical bonds in such a way that the material maintains its robustness and keeps its structure, but is weak enough not to block antibacterial activity [11].

In the former described mechanism, antibacterial properties stem not only from the release of bioactive metal ions but also from the organic linkers, giving rise to a combined synergetic antibacterial effect in the media. Another critical factor in promoting its antibacterial properties is the particle size of the MOF [12]; decreasing its particle size to the nanoscale range results in a considerable enhancement of the surface area, allowing for a greater numbers of interactions with its surrounding, and even prompting its internalisation into the cells.

The multifunctional characteristics of copper make it an ideal candidate for application in fields as wide as from catalysis to adsorption [4,13]. Cu^2+^ has recognised antifouling, antifungal and antibacterial properties; it is also relatively affordable, abundant and well-known for eradicating bacterial infections [14,15,16,17]. It has been widely used in clinics in particular, where multiple applications of copper-based sterilising materials have been found [1,11].

Among the copper-based MOF materials—for example, HKUST-1 and MOF-199—many have been extensively studied in the field of bactericidal materials due to their simple synthetic route and the low toxicity of their ligands [18]. Several approaches for immobilising MOFs into various substrates have been carried out over the last few years with the idea of developing new materials with promising properties [2,19]. The application spectrum is broad: from textiles that could be used as wound dressings that inhibit bleeding and promote healing, to coatings that prevent bacterial adhesion to surfaces to avoid biofilm formation [19]. In this line, Rodríguez et al. [11,20] reported the immobilisation of Cu-BTC on cellulose fibres, exhibiting good antibacterial activity for *Escherichia coli* (*E. coli*) [20]. The immobilisation was performed by exposing cellulosic substrates to Cu-BTC MOF precursors to achieve in situ synthesis of the material in a basic media. The antibacterial activity, in this case, arises from gradual degradation and Cu^2+^ liberation, inducing damage to the bacterial envelope [21].

Another possible shaping and immobilising procedure can be packing MOFs into pellets, monoliths or membranes. As MOFs generally yield insoluble and non-dense products, they tend to display problems when incorporated into devices as they can blow off and contaminate pipes easily in charge/discharge cycles. Thus, bulk-material processing has become a mandatory procedure for implementing MOFs in the industry. The selection of the shaping technique depends on the textural properties of the chosen material. Generally, the shaped material must keep or enhance its mechanical strength and it must be a simple and cost-effective procedure [22,23].

Even though shaping and processing MOFs for specific applications is still at an early stage, efforts are dedicated to bringing MOFs towards real-world applications [24,25,26,27]. Thus, apart from shaping, it has become mandatory to test processed materials under the humidity, applied-pressure conditions and high operating temperatures that can be encountered in industrial processes. In this line, Figueira et al. [28] reported a work where they presented a simple and inexpensive method for immobilising MOFs in the form of pellets and membranes and studied the stability of processed materials in terms of their resistance to different temperatures and humidity to simulate possible industrial operating conditions. They performed these studies in Cu-BTC and MOF-74 MOFs, motivated by the well-known capacity of these materials to adsorb harmful gases such as CO_2_ and due to the possible competition of ambient water molecules with the gases that are supposed to be captured in the porous network [29].

In this work, we report a novel three-dimensional copper-based MOF composed of the bioactive 3,4-dihydroxybenzoic acid ligand. The structure displays the formula [Cu_3_L_2_(DMF)_2_]_n_, (**1**), where H_3_L corresponds to the organic linker. Considering the antibacterial activity of Cu^2+^ ions and that the organic linker composing the MOF—which exhibits well-known performance against pathogenic bacteria [30]—**1**’s antibacterial activity has been studied and compared with the free ligand and Cu^2+^ as controls. Additionally, following the immobilisation approach of the material reported by Figueira et al. [28], **1** has been shaped into pellets and membranes, and its behaviour under different thermal and moisture conditions has been studied in processed materials. Additionally, the microporous structure of **1** enabled us to test the CO_2_ adsorption of the material following the Yang et al. [29] strategy, where the surface area and CO_2_ adsorption of Cu-BTC was improved via a solvent-exchange procedure.

## 2. Results and Discussion

### 2.1. Crystal Structure Details

**Figure 1:**
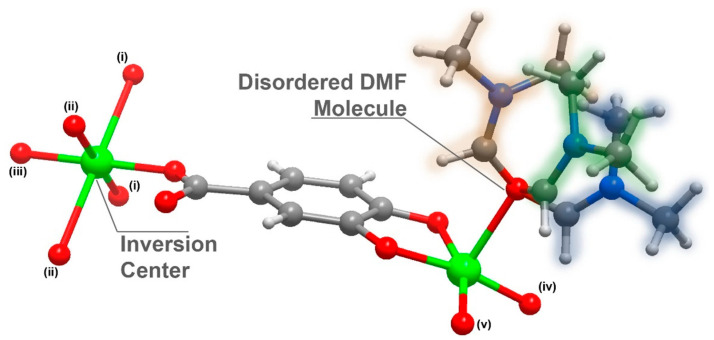
Schematic representation of the asymmetric unit in [Cu_3_L_2_(DMF)_2_]_n_ (**1**), emphasizing the disorder present in the coordinated DMF molecule and the inversion centre present in the Cu2 atom. Metal centre coordination environments were generated using the symmetry transformation (i) x + 3/2, y + 1/2, −z + 1/2; (ii) x + 1/2, −y + 3/2, z + 1/2; (iii) −x + 2, −y + 2, −z + 1; (iv) x − 1/2, − y + 3/2, z − 1/2; (v) – x + 1, y, − z + 1/.

Compound **1** was further analysed by single-crystal X-ray crystallography and its structure unveiled was as [Cu_3_L_2_(DMF)_2_]_n_·(DMF = *N*, *N*-dimethylformamide); **1** crystallized in the centrosymmetric space group *C*2/*c* with an asymmetric unit composed of 1.5 Cu^2+^ cations (half of the Cu^2+^ is located in an inversion point), a L^3−^ organic linker and a highly disordered DMF molecule (Figure 1).

**Figure 2:**
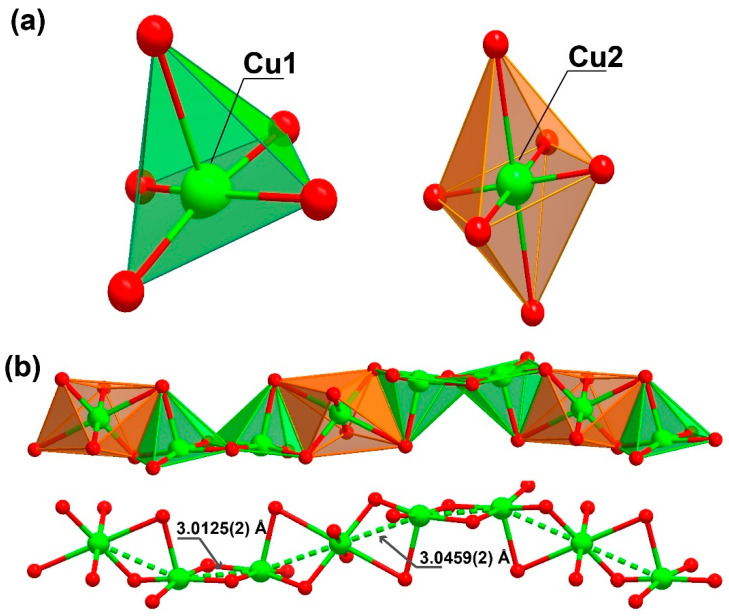
Representation of the (**a**) coordination environment present for the two crystallographically independent Cu^2+^ cations, exhibiting a square pyramid and a octahedral coordination sphere (for Cu1 and Cu2, respectively) and (**b**) the inorganic chain with a “ABBA” connectivity between the metal centres extending along the (011) plane present in [Cu_3_L_2_(DMF)_2_]_n_ (**1**).

The organic linker 3,4-dihydroxybenzoate was fully deprotonated and showed coordination to the metallic centre, both from the carboxylate moiety as well as from the phenoxide groups. The oxygens in the carboxylic group coordinated to two metal centres in a simple κ^−1^-*O* coordination mode, while the oxygens from the phenoxide groups coordinated in a μ_2_-*O,O* and κ^−2^-*O* coordination mode, coordinating to three metal centres. The Cu1 atom displayed a penta-coordinated environment ({CuO_5_}, Figure 2a) being linked to three ligands and a DMF molecule. Concretely, the CuO_5_ environment was composed of the coordination of two phenoxide moieties of one ligand (one oxygen worked as a bridge between two neighbouring Cu1 centres and the other between a Cu1 and a Cu2 centre) and an additional phenoxide moiety that corresponded to another linker molecule (which also connected two Cu1 metals, with *d*_Cu1···Cu1_ = 3.0125(2) Å), as well as the coordination of one donor atom belonging to the carboxylate moiety of another ligand molecule. The surrounding of Cu1 was completed by coordinating an additional oxygen atom belonging to a DMF solvent molecule, which acted as a direct bridge between a Cu1 and a Cu2 metal centre.

Cu2, instead presents with a hexa-coordinated environment ({CuO_6_}, Figure 2a), being linked to four ligands and two symmetry-related DMF molecules. When the CuO_6_ surrounding was examined in detail, two of the oxygen atoms belonged to phenoxide moieties of two distinct linker molecules, which acted as a nexus between the Cu1 and Cu2 centres (*d*_Cu1···Cu2_ = 3.0459(2) Å), in addition to the coordination of another two oxygen donor atoms belonging to a carboxylate moiety of two symmetry-related linkers. The coordination sphere was then completed by two oxygen atoms from the DMF molecules. Continuous shape measurements (CShMs) [31] revealed that the Cu1 and Cu2 atoms built different polyhedra, with Cu1 and Cu2 resembling a square pyramid (SPY-5) and octahedron polyhedra (OC-6), respectively (see Tables S1 and S2 for more detailed information).

**Figure 3:**
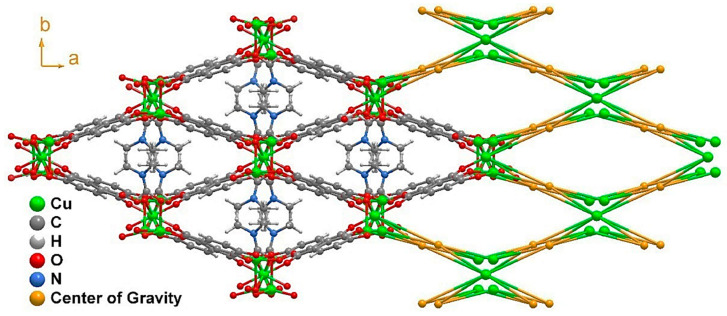
Structural packing of [Cu_3_L_2_(DMF)_2_]_n_ (**1**) as viewed in the (001) direction, alongside its topological view. The disorder present in the DMF molecule was removed for clarity.

This high level of coordination led to the formation of an inorganic chain extending along the (011) plane (Figure 2b). Taking into account the connectivity achieved among these SBUs, it may be considered an ‘‘ABBABBA’’ model, considering A as Cu2 and B as Cu1. These inorganic chains are in turn “connected” by the organic linker, forming a porous 3D structure (Figure 3 and Figure S1).

Considering the connectivity of all the metals and ligands, the resulting structure possessed a previously non-described topology (Figure 3) that can be simplified by the (4^2^·8^2^·10^2^)(4^3^)_2_(4^4^·6^3^·8^3^)_2_ point symbol [32]. The growth of this structure along the *c* axis left narrow microchannels that were occupied by disordered DMF molecules displaying a pointing-out disposition in the pores and taking advantage of the whole pore cavity; thus, no solvent-accessible volume was left according to the geometrical calculations performed with the PLATON-v1.18 program [33]. This is also supported by the thermogravimetric analysis (TGA) and X-ray performed (Figures S2–S5).

### 2.2. Processing into Pellets and Membranes

Following the approach of Figueira et al., [28] the bulk material was processed into pellets and membranes to explore simple and cost-effective processing techniques [28]. A homemade extrusion system allowed for the compression of **1**, forming a pellet; for the purpose of supporting cohesion to the bulk material, water was employed as a binding agent. The aforementioned procedures are described in detail in the Supplementary Material. With the materials shaped, moisture and temperature stability studies were carried out and the stability of the processed material was determined by powder X-ray diffraction (PXRD).

### 2.3. Moisture Stability

The powder X-ray diffraction patterning performed in **1** demonstrated that the pristine MOF, as well as the shaped materials (pellets and membranes), kept its stability under high relative humidity (98% RH) conditions for 72 h. In the case of the membrane-immobilised material of **1**, despite the amorphous nature of the polysulphone (PSF) polymeric matrix, the reflexions from **1** were still present and well-defined. The PXRD results (Figure S6) show that compound **1** was stable and kept its structure after being processed into pellets or membranes and being exposed for 72 h to 98% RH. Nonetheless, in the additional tests related to the solvent-exchange procedure (where we examine the material’s stability upon being submerged in methanol, ethanol and water), we appreciated that when the material (**1**) was in direct contact with water, it evolved into another crystalline phase; however, prolonged exposure to humidity did not provoke a material transformation and it remained stable.

### 2.4. Temperature Stability

After performing humidity tests, the pellets were tested against temperature cycles. Four heating and cooling-down cycles (from 125 °C to room temperature—RT) were conducted and the shaped materials were characterised by PXRD analysis. A photograph taken after each cycle gave an idea of the integrity of the pellet (Figure S7). After the second and fourth cycles, PXRD analyses were conducted in coated and uncoated pellets. Coated and uncoated pellets of **1** exhibited excellent resistance to humidity and subsequent temperature-cycles, maintaining the pellet’s integrity and not suffering from humidity-derived structural transformation. Nonetheless, it must be admitted that coated pellets kept their integrity better as the resistance was supported by the polymer.

### 2.5. Adsorption Studies

#### 2.5.1. Water Adsorption

Water-vapour adsorption isotherms were measured at 25 °C in the bulk of **1** to ascertain the amount of water adsorbed at different relative humidities (2–98%). According to the results, the as-synthesised (AS) compound **1** presented with a very low variation in mass change—precisely 1.5%—which could be indicative of monolayer-type adsorption in the surface of the MOF. Nonetheless, when the solvent-exchange procedure was carried out (stirring 100 mg of compound **1** in 4 mL of EtOH for 16 h) by thermogravimetric analysis (TG) and PXRD, it was deduced that the material remained stable and it appeared that the coordinated DMF molecules were replaced by EtOH molecules (see Figure S8 for more detailed information). In agreement with this, the water adsorption isotherm showed a three-times higher mass change than the as-synthesised compound **1**, exhibiting an uptake of 6%. However, compound **1** exhibited low uptake even after the solvent-exchange procedure, which could be due to the hydrophobic character of the MOF.

#### 2.5.2. Gas Adsorption Capacity

Subsequently, to assess Cu-MOF porosity, we studied its adsorption capacity toward gases. First, we performed adsorption isotherm studies on the as-synthesised material **1** towards N_2_ and CO_2_ adsorbates at 77 K and 273 K, respectively, after an outgassing step at 170 °C for 6 h (Figure S9). Regarding N_2_ adsorption, Cu-MOF loaded 7.9 cm^3^/g (0.4 mmol/g) at the maximum relative pressure (P/P_0_ = 1). This low N_2_ uptake capacity resulted in a low surface area value, 4 m^2^/g—characteristic of non-porous materials—as calculated using the Brunauer–Emmett–Teller (BET) equation. Focusing on CO_2_ adsorption, its load was also low, barely surpassing 5 cm^3^/g (0.2 mmol/g) at a relative pressure of 0.03.

**Figure 4:**
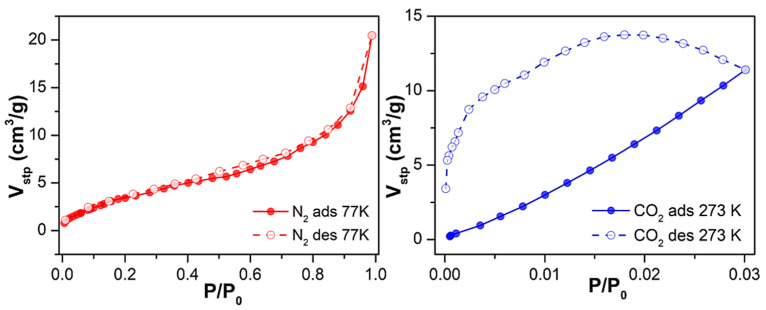
N_2_ and CO_2_ adsorption and desorption isotherms at 77 K and 273 K of **1** upon outgassing at 170 °C for 6 h after solvent exchange with EtOH.

Considering the relatively low uptake for N_2_ and CO_2,_ we decided to proceed with the solvent exchange procedure as it was performed for the water-adsorption studies. Similarly, compound **1** was suspended in ethanol for 16 h, and the sorption studies were repeated. In the latter case, sample outgassing was performed for the as-synthesised material, and the adsorption capacity was evaluated for N_2_ at 77 K and CO_2_ at 273 K (Figure 4). Under the conditions mentioned earlier, **1** exhibited a 2.5-fold increased maximum N_2_ loading of 20 cm^3^/g (0.9 mmol/g), indicating that the solvent exchange strategy had a positive, although limited, effect on its mesoporosity. This was reflected in its overall surface area, as the BET surface almost quadrupled from 4 m^2^/g to 15 m^2^/g.

Regarding CO_2_ uptake, adsorption in the micropores increased (up to 11 cm^3^/g at P/P_0_ = 0.03, corresponding to 0.5 mmol/g), exhibiting—according to the Dubining-Astakhov equation—a micropore volume of 0.5 cm^3^/g, significantly larger than the 0.15 cm^3^/g displayed by the as-synthesised material. The obtained results are comparable and in line with work reported in the literature so far [34,35,36]. It can be highlighted that in the work of Güçlü et al. they reported a family of transition metal and oxalamide-functionalized-based MOFs displaying CO_2_ adsorption; in particular, the Cd-OATA MOF exhibited 0.51 mmol/g adsorption at 298 K.

### 2.6. Antibacterial Activity

**Figure 5:**
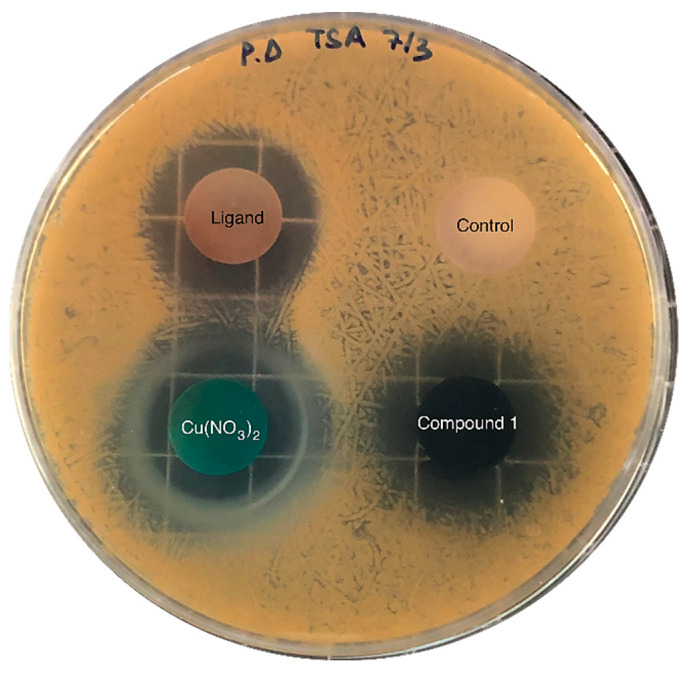
Diffusion assays showing the inhibitory activity against *S. aureus* of copper nitrate, H_3_L (ligand) and compound **1**. The negative control sample—without inhibitory activity—contained calcium phosphate, which was also used as a supporting material in the rest of the samples (25 mg of active compound and 75 mg of calcium phosphate).

The inhibitory activity of the samples was assessed against pathogenic gram-positive *Staphylococcus aureus* (*S. aureus* bacteria), which causes a wide variety of clinical diseases [37]; the results are shown in Figure 5. Copper nitrate, H_3_L and compound **1** inhibited the bacteria’s growth in a media that was favourable for the growth of the pathogen. Table 1 shows the area of inhibition generated around each of the solid samples after 24 h incubation. As can be observed, both samples—the copper salt and compound **1**, which contained a similar mass of Cu (Table 1)—exhibited practically the same area of inhibition. Thus, *S. aureus* pathogenic growth can likely be inhibited upon MOF dissolution and subsequent Cu^2+^ release, which further diffuses through the agar; the ligand’s release can also partially inhibit its growth. These results suggest that the inhibitory activity of Cu and the ligand remained practically unaltered after the formation of the MOF.

The reported results are in line with the work reported by Lee et al. [38], where they investigated the antibacterial activity derived from NO-releasing Cu-BTC by the disk diffusion method in six bacterial strains. Regarding the obtained results for the *S. Aureus* Gram-positive strain, the inhibition area was 414.2 ± 18.4 mm^2^ for Cu-BTC and 376.8 ± 21.6 mm^2^ for NO⊂Cu-BTC.

## 3. Materials and Methods

### 3.1. Preparation of Complexes

All chemicals were reagent grade and were used as obtained.

#### Synthesis of [Cu_3_L_2_(DMF)_2_]_n_

- General procedure for synthesising single crystals: 0.010 g (0.065 mmol) of 3,4-dihydroxybenzoic acid (L) organic linker was dissolved in 0.5 mL of DMF. In a separate vial, 0.010 g (0.0434 mmol) of Cu(NO_3_)_2_·2.5H_2_O was dissolved in 0.5 mL of distilled water. After the ligand and metal dissolution, 0.5 mL of H_2_O was added to the ligand solution and 0.5 mL of DMF to the metal solution. The metal solution was added dropwise to the ligand solution with magnetic stirring. The resulting greenish-turquoise solution was poured into a screw-capped vial (6 mL) and introduced to the oven at 95 °C for 6 h, giving rise to dark-brown ribbon-shaped single crystals. Single-crystal X-ray structure determination, FT-IR (Figure S10), elemental analysis (EA; Table S3) confirmed the general formula [CuL_2_(DMF)_2_]_n_.
- General procedure for the scale-up synthesis: 0.2 g (1.2 mmol) of 3,4-dihydroxybenzoic acid ligand and 0.2 g (0.868 mmol) of Cu(NO_3_)_2_·2.5H_2_O were weighed and dissolved in 3 mL DMF/3 mL H_2_O solvent mixture. This greenish-turquoise solution was placed in a microwave and heated at 95 °C for an hour to obtain around 85 mg of Cu MOF (yielding ~75%). PXRD confirmed the purity of the product. Data related to the EA, FTIR and TG can be found in the Supplementary Material. SEM images of compound **1** are shown in Figure S11.

### 3.2. X-ray Diffraction Data Collection and Structure Determination

Details of the structure determination and refinement of compound **1** are summarised in Table 2 and Table S4. Crystallographic data for the structures reported in this paper have been deposited with the Cambridge Crystallographic Data Center as a supplementary publication—CCDC 2195988. Power X-ray diffraction is shown in Figure S12. Moreover, compound 1 stability was examined in various solvents (Figure S13).

### 3.3. Inhibitory Activity against Staphylococcus Aureus (S. aureus)

The pathogenic strains of Staphylococcus Aureus (CECT 976, *S. aureus*) were supplied by the Colección Española de Cultivos Tipo (CECT). The pathogenic strain was grown in tryptic soy broth (TSB No2, Sigma-Aldrich) at 30 °C, following the supplier’s recommendations.

The inhibitory activity of copper nitrate, the ligand and compound **1** was evaluated by agar diffusion assays. This assay was carried out as follows: 0.1 mL of an overnight culture of *S. aureus* was spread on Petri dishes containing TSA (containing 3 % *v*/*v* of TSB and 1.5% *w*/*v* agar). Then, pellets of each sample were placed on agar plates containing the pathogenic bacteria and incubated at 30 °C—the pathogen’s optimal temperature. After 24 h of incubation, the inhibition zones were imaged and compared. Pellets of 100 mg were prepared by mixing 75 mg of calcium phosphate and 25 mg of the sample (copper nitrate, the ligand or compound **1**, respectively) and then pressed using a compact hydraulic press at 10 tons. A 100 mg calcium phosphate pellet was also prepared and analysed as a negative control. Inhibition experiments were performed in triplicate. The inhibition area was measured with ImageJ software; it was obtained by subtracting the area of the pellet from the total area. Data are shown as mean values and the corresponding standard deviation (SD).

### 3.4. Material Shaping: Processing into Pellets and Membranes

Pellets: 100 mg of compound **1** was weighed and 100 µL of water was added to yield a malleable paste. Afterwards, the paste was transferred into a syringe and compressed by applying heat to eliminate excess binding agent. For increased endurance, pellets of **1** were submerged for 2 s in a solution containing 300 mg of polysulphone (PSF) in CH_2_Cl_2_. Solvent evaporation gave place to a pellet of **1** covered by a transparent film of PSF.

Membranes: Compound **1** was also immobilised in polymeric membranes. This material was shaped according to the following procedure: 400 mg of polysulphone (PSF) was weighed and dissolved in 5 mL of dichloromethane (CH_2_Cl_2_). To this dense solution, 100 mg of compound **1** were added and stirred for 30 min. The remaining viscous solution was then cast in a glass petri dish and left unstirred at ambient conditions until the complete evaporation of the solvents. Note that the membrane preparation conditions were optimised through essays containing various MOF-to-polymer ratios to achieve a balanced coverage homogeneity and mechanical stability.

### 3.5. Pellet and Membrane Moisture Stability Tests

After compound **1** was shaped into pellets and membranes, the stability of these processed materials was examined against possible operating industrial conditions such as moisture and temperature cycles. So, first, pellets and membranes of **1** were treated for 72 h in a desiccator containing a K_2_SO_4_-saturated solution, which simulated 98% relative humidity (RH) [39]; subsequently, the moisture stability was checked. Afterwards, pellets were treated with temperature cycles. In all cases, the stability of the processed material was determined by PXRD.

## 4. Conclusions

We have synthesised a novel copper MOF based on 3,4-dihydroxybenzoic acid as a ligand shaped by a simple and non-expensive processing method. Furthermore, we checked the shaped material’s stability by means of temperature and ambient moisture levels to simulate possible industrial operating conditions, exhibiting good mechanical stability and integrity in pelletised materials and membranes. Additionally, the microporous structure of the three-dimensional MOF allowed us to study the adsorptive capacity of **1**, displaying almost negligible CO_2_ uptake in the as-synthesised material. However, the solvent exchange procedure allowed a partial replacement of coordinated DMF molecules with ethanol, maintaining its structural stability and easing the material’s activation by replacing a more volatile solvent. Consequently, sorption isotherms were repeated, exhibiting an uptake of 0.54 mmol/g. Finally, motivated by the known antibacterial capacity of the Cu^2+^ and 3,4-dihydroxybenzoic acid ligands, we decided to study the antibacterial activity of compound **1** and its precursors towards pathogenic *S. aureus* bacteria by the disk-diffusion method, exhibiting that the antibacterial activity of both the Cu^2+^ and 3,4-dihydroxybenzoic acid ligand remained practically unaltered after the formation of the compound.

## Figures and Tables

**Table 1:** Area of inhibition (cm^2^) of the compound against *S. aureus*. Values are shown as mean ± standard deviation (SD, n = 3). The mass of Cu and H_3_L in each sample is also provided.

Compound	Inhibition Area (cm^2^)	Mass of Cu (mg)	Mass of Ligand (mg)
H_3_L	3.6 ± 0.3	-	25
Cu(NO_3_)_2_	4.2 ± 1.7	6.8	-
Compound **1**	4.1 ± 0.8	6.9	10.7

**Table 2:** Crystallographic data and structure refinement details of compound **1**.

Compound	1
Formula	C_10_H_10_Cu_1.50_NO_5_
Formula weight	319.50
Temperature/K	150 (2)
Crystal system	Monoclinic
Space group	*C2/c*
*a*/Å	20.2893 (16)
*b*/Å	8.6194 (7)
*c*/Å	16.7337 (11)
*α*/º	90.0
*β*/º	126.632 (2)
*γ*/º	90.0
Volume/Å^3^	2348.4 (3)
*Z*	8
*µ(*Mo Kα*)*/mm^−1^	2.75
Crystal type	Red plate
Crystal size/mm	0.16 × 0.14 × 0.06
*θ* range (°)	3.42–25.35
Index ranges	−24 ≤ *h* ≤ 22 −10 ≤ *k* ≤ 10 −20 ≤ *l* ≤ 20
Collected Reflections	17154
Independent Reflections	2141 (*R*_int_ = 0.023)
Completeness to *θ =* 25.24	99.6%
Final *R* indices [*I* > 2σ(*I*)]	*R*1 = 0.0354 *wR*2 = 0.0964
Final *R* indices (all data)	*R*1 = 0.0386 *wR*2 = 0.0984
Largest diff. peak and hole /eÅ^−3^	0.66 and −0.99

*^a^*$R1=\sum \left|\left|F_{o}\right|-\left|F_{c}\right|\right|/\sum \left|F_{o}\right|$, *^b^*$wR2=\sqrt{\sum \left[w{\left(F_{o}^{2}-F_{c}^{2}\right)}^{2}\right]/\sum \left[w{\left(F_{o}^{2}\right)}^{2}\right]}$, *^c^*$w=1/\left[\sigma^{2}\left(F_{o}^{2}\right)+{\left(mP\right)}^{2}+nP\right]$ where $P=\left(F_{o}^{2}+2F_{c}^{2}\right)/3$.

## Data Availability

The data that support the findings of this study are available from the corresponding author upon reasonable request.

## Supplementary Materials

The following supporting information can be downloaded at: https://www.mdpi.com/article/10.3390/molecules27228073/s1, Table S1: Table of the continuous Shape Measurements for the CuO_5_ coordination environment; Figure S1: View along *a*, *b* and *c* crystallographic axis, the corresponding topological representation and the view of “ABBA..” SBU; Table S2: Table of the continuous Shape Measurements for the CuO_6_ coordination environment; Figure S2: Figure of TG/DTA analysis of as synthesised compound **1**; Figure S3: Figure of TG/DTA analysis of compound **1** performed after solvent exchange with EtOH during 16 h (left side) and experimental PXRD for complex **1** before and after solvent exchange with EtOH (right side); Table S3: Elemental analysis of compound **1**; Table S4: Selected bond lengths (in Å) and bond angles (in °) for the Cu^2+^ coordination environments present in [Cu_3_L_2_(DMF)_2_] (**1**); Figure S4: Thermal evolution of as synthesised compound **1**; Figure S5: Comparison between the PXRD transformed phase of compound **1** obtained from thermal evolution at 190 °C and the phase obtained soaking compound **1** in H_2_O during 16 h; Figure S6: Powder X-ray diffractograms of the studied materials, as-synthesized and after processing into pellets and membranes subsequent moisture treatment; Figure S7: Powder X-ray diffractograms of the studied materials, as-synthesized and after processing into pellets after temperature-cycles; Figure S8: Water adsorption isotherm of compound **1** as synthesised (AS) and after solvent-exchange with EtOH for 16 h; Figure S9: N_2_ and CO_2_ adsorption at desorption isotherms at 77 K and 273 K of **1**, upon outgassing at 170 °C for 6 h; Figure S10: Infrared spectra of the ligand and compound **1**; Figure S11: SEM images of compound **1**; Figure S12: Figure of the pattern matching analysis and experimental PXRD for complex **1**; Figure S13: Experimental PXRD for complexes **1** after being soaked for 16 h in several solvents. References [40,41,42,43,44,45,46,47,48] are cited in the supplementary materials.

## Sample Availability

Samples of the compounds … are available from the authors.
