# Supplementary material for: Adsorptive Capacity, Inhibitory Activity and Processing Techniques for a Copper-MOF Based on the 3,4-Dihydroxybenzoate Ligand
Source: Molecules. 2022 Nov 21;27(22):8073. doi: 10.3390/molecules27228073 (PMC9695574; doi:10.3390/molecules27228073)
Supplement: Supplementary file 1 [file molecules-27-08073-s001.zip › molecules-2017689-supplementary.pdf]

Supporting Information for:

Adsorptive capacity, Inhibitory Activity and Processing Techniques for a Copper-MOF Based on the 3,4-Dihydroxybenzoate ligand

Estitxu Echenique-Errandonea [1], Sara Rojas [2], Víctor Karim Abdelkader-Fernández [2], Manuel Pérez-Mendoza [2], Ricardo F. Mendes [3], Paula Barbosa [4], Filipe Figueiredo [4], Flávio Figueira [3], Filipe A. Almeida Paz [3], José Manuel Delgado-López [2], Antonio Rodríguez-Diéguez [2,*] and José Manuel Seco [1,*]

1 Departamento de Química Aplicada, Facultad de Química, Universidad del País Vasco UPV/EHU, Paseo Manuel Lardizabal, N° 3, 20018, Donostia-San Sebastián, Spain
2 Departamento de Química Inorgánica, Facultad de Ciencias, Universidad de Granada, Av. Fuentenueva S/N, 18071 Granada, Spain
3 Department of Chemistry, CICECO – Aveiro Institute of Materials, University of Aveiro, 3810-193 Aveiro, Portugal
4 Department of Materials & Ceramic Engineering, CICECO – Aveiro Institute of Materials, University of Aveiro, 3810-193 Aveiro, Portugal
* Correspondence: antonio5@ugr.es, josemanuel.seco@ehu.eus

Table of Contents:

Experimental Section 3

General Instrumentation 3

Chemical characterization of compounds 4

Continuous Shape Measurements 4

Elemental analysis 5

Additional views of the structure 5

Thermal analysis 6

Thermal evolution 7

MOF processing: Transformation into pellets and membranes 9

Moisture stability 9

Temperature stability 10

Water adsorption analysis 11

Adsorption properties 12

FT-IR spectroscopy 13

Scanning Electron Microscopy 14

Powder X-ray diffraction analysis 15

Solvent stability 15

Selected bond lengths and angles data 16

References 17

Experimental Section

General Instrumentation

Elemental analyses (C, H, N) were performed on a Leco CHNS-932 microanalyser.

IR (ATR) spectra of products were recorded in a FT-IR Bruker Alpha spectrometer. IR spectra of Ligand and Compound **1** were recorded in the region 400–4000 cm^{-1} on a Nicolet 6700 FTIR (Fourier transform infrared) spectrophotometer (Thermo Phisher Scientific, TX, USA) with samples as KBr disks.

Thermogravimetric analysis (TG/DTA) were performed on a TG-Q500 TA Instruments thermal analyser from room temperature to 800 °C under a synthetic air atmosphere (79% N_2/21% O_2) at a heating rate of 10 °C min^{-1}.

X-ray powder diffraction (XRPD) patterns were collected at 25 °C on a Phillips X'PERT powder diffractometer with Cu-Kα radiation (λ = 1.5418 Å) over the range 5 < 2θ < 50° with a step size of 0.02° and an acquisition time of 2.5 s per step. Indexation of the diffraction profiles were made by means of the FULLPROF program (pattern- matching analysis) based on the space group and the cell parameters found by single crystal X-ray diffraction [1].

Scanning electron microscopy (SEM) images were acquired using either a Hitachi S4100 field emission gun tungsten filament instrument working at 25 kV or a high-resolution Hitachi SU-70 working at 4 kV. Samples were prepared by deposition on aluminium sample holders followed by carbon coating using an Emitech K950X carbon evaporator. EDS (energy dispersive X-ray spectroscopy) data and SEM mapping images were recorded using the latter microscope working at 15 kV and using either a Bruker Quantax 400 or an Esprit 1.9 EDS microanalysis system.

The **water vapour adsorption isotherms** were collected in a Dynamic Vapor Sorption apparatus from Surface Measurements Systems, using N_2 as the carrier gas (Air Liquide Alphagaz, less than 3 ppm H_2O, total flow of 200 sccm). Dry aliquots (16 mg) were loaded in a steel pan and suspended in the measuring chamber. The experiment started with a 2 h pre-treatment at 125 °C, to completely dry the sample, followed by the isotherm at 25 °C with increasing and decreasing RH steps from 0 to 98 %. Each humidity step was kept until the rate of change of mass per fixed time (dm/dt) was lower than 0.002 %, for a period of at least 10 min.

Single Crystal X-ray diffraction: Single crystals of compound **1** were manually harvested from the crystallization vials and immersed in highly viscous FOMBLIN Y perfluoropolyether vacuum oil (LVAC 140/13, Sigma-Aldrich) to avoid degradation caused by the evaporation of the solvent [2]. Crystals were mounted on either Hampton Research CryoLoops or MiTeGen MicroLoops, typically with the help of a Stemi 2000 stereomicroscope equipped with Carl Zeiss lenses. X-ray diffraction data for compound **1** was collected at 150(2)K on a Bruker D8 QUEST equipped with Mo Kα sealed tube (λ = 0.71073 Å), a multilayer TRIUMPH X-ray mirror, a PHOTON 100 CMOS detector, and an Oxford Instruments Cryostrem 700+ Series low temperature device. Diffraction images were processed using the software package SAINT+ [3], and data were corrected for absorption by the multiscan semi-empirical method implemented in SADABS 2016/2 [4]. All structures were solved using the algorithm implemented in SHELXT-2014/5 [5], which allowed the immediate location of almost all of the heaviest atoms composing the molecular unit of the four compounds. The remaining missing and misplaced non-hydrogen atoms were located from difference Fourier maps calculated from successive full-matrix least-squares refinement cycles on F^2 using the latest SHELXL from the 2018/3 release [6]. All structural refinements were performed using the graphical interface ShelXle [7]. Hydrogen atoms bound to carbon and oxygen were placed at their idealized positions using appropriate *HFIX* instructions in SHELXL: *43* (aromatic carbon atoms and =CH– groups from the solvent molecule) and *33* (for terminal methyl groups). These hydrogen atoms were included in subsequent refinement cycles with isotropic thermal displacements parameters (U_{iso}) fixed at 1.2 or 1.5×U_{eq} of the parent non-hydrogen atoms, respectively. The last difference Fourier map synthesis showed the highest peak (0.69 eÅ^{-3}) and the deepest hole (-0.99 eÅ^{-3}) located at 1.10 and 0.03 Å from H11B and C22, respectively. Structural drawings have been created using the software package Crystal Impact Diamond [8].

Chemical characterization of compounds

Continuous Shape Measurements

CShMs for the coordination environment of compounds **1**. The lowest SHAPE values for each ion is shown highlighted in grey, indicating best fits [9].

Table S1. Table of the continuous Shape Measurements for the CuO_5 coordination environment.

PP-5	D_{5h}	Pentagon
vOC-5	C_4	Vacant octahedron (Johnson square pyramid, J1)
TBPY-5	D_{3h}	Trigonal bipyramid
SPY-5	C_{4v}	Square pyramid
JTBPY-5	D_{3h}	Johnson trigonal bipyramid (J12)

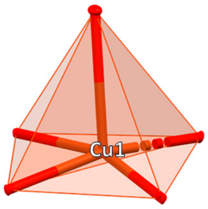

Complex 1	PP-5	vOC-5	TBPY-5	SPY-5	JTBPY-5
**Cu1**	29.740	1.653	6.289	**1.062**	9.155

Table S2. Table of the continuous Shape Measurements for the CuO_6 coordination environment.

HP-6	D_{6h}	Hexagon
PPY-6	C_{5v}	Pentagonal pyramid
OC-6	O_h	Octahedron
TPR-6	D_{3h}	Trigonal prism
JPPY-6	C_{5v}	Johnson pentagonal pyramid J2

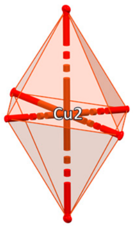

Complex 1	HP-6	PPY-6	OC-6	TPR-6	JPPY-6
**Cu2**	29.385	28.101	**2.417**	17.463	30.372

Elemental analysis

Table S3. Elemental analysis of compound **1**

Compound	Formula	Molecular weight	Calc.	Found.
**1**	$C_{10}H_{10}Cu_{1.5}NO_5$	319.5	C: 37.59; H: 3.15; Cu: 29.83; N: 4.38; O: 25.04	C: 37.63; H: 3.20; Cu: 29.80; N: 4.37; O: 25.11

Additional views of the structure

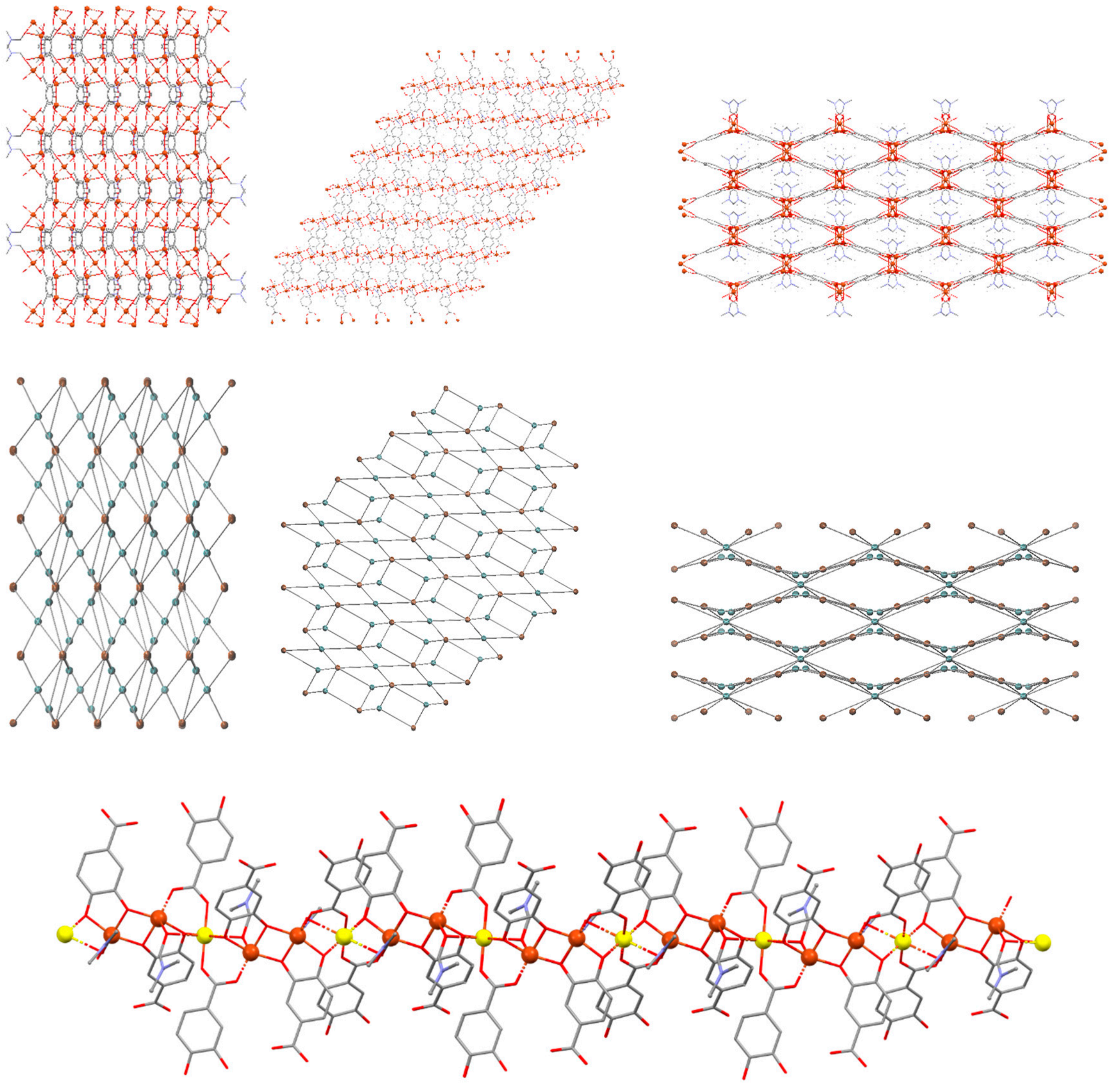

Figure S1. View along *a*, *b* and *c* crystallographic axis, the corresponding topological representation and view of "ABBA.." SBU.

Thermal analysis

Thermogravimetric analyses performed over polycrystalline sample in compound **1** enabled checking the stability of the product. TG curves has been collected for compound **1** for the as synthesised compound **1** and after solvent exchange with EtOH. In stability section, we saw that material remains stable after being soaked in EtOH and being this latter solvent more volatile than the solvents used in the synthesis of the material, we tried to accomplish solvent exchange procedure as an approach to replace solvent molecules (dimethylformamide and water molecules) to ease material activation to posteriorly analyse its adsorptive-capacity. For this purpose, as for stability tests, compound **1** was suspended in EtOH for 16 h, left solvent evaporated and carried on thermal analysis. Powder X-ray diffraction confirmed that **1** remains stable after solvent exchange with MeOH as it can be seen in Figure S.

The thermal behaviour of the as synthesised $[Cu_3L_2(DMF)_2]_n$, compound **1**, exhibits two main regions. The first weight of loss was progressive and went from ambient temperature to 200 ^{o}C and corresponded to the loss of the DMF coordinated molecules. Above this temperature, there is an abrupt loss corresponding to the complete collapse of the crystal structure. From the shape of the TG curve, it seems that solvent molecules stabilise the structure and their removal promote crystal structure decomposition. In the final step, as a consequence of the decomposition of the organic content metal oxide is obtained.

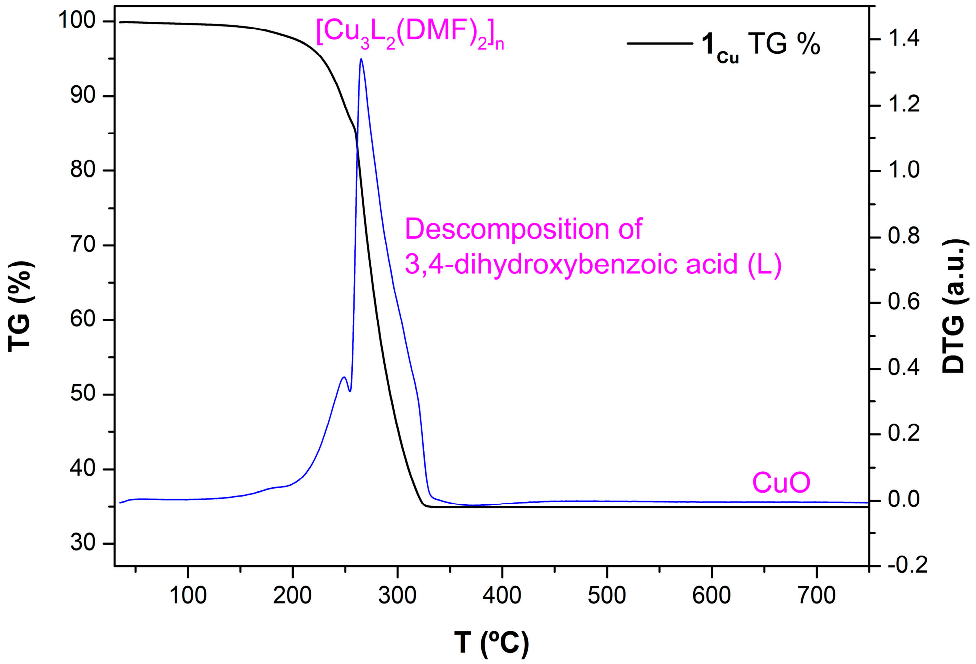

Figure S2. Figure of TG/DTA analysis of as synthesised compound **1**.

TG curve performed after solvent exchange with EtOH exhibits different pattern in comparison to the previous curve obtained with as synthesised compound **1** material. In this case, after solvent exchange procedure, the weight of loss occurs in three main regions. The first region comprises from the room temperature to 150 ^{o}C and after an initial loss of weight occurring from 30 to 70 ^{o}C a plateau is obtained around 80 ^{o}C which goes up to 150 ^{o}C. Then, from 150 ^{o}C to 200 ^{o}C a second loss of weight is visible in the spectra before the last abrupt loss corresponding to compound **1** degradation which occurs at 250 ^{o}C. Above this temperature, CuO is obtained as final residue. The plateau obtained in the at around 80 ^{o}C and the increase of final-residue percentage (in around 8 %) can be indicative of solvent molecules being replaced by EtOH, being this solvent-exchange strategy a possible option to promote material activation at lower temperatures.

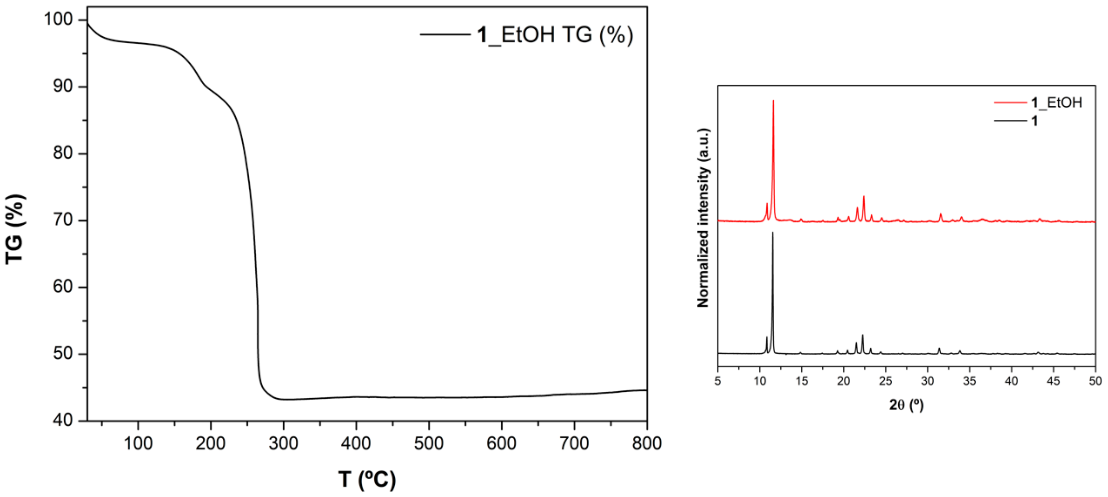

Figure S3. Figure of TG/DTA analysis of compound **1** performed after solvent exchange with EtOH during 16 h (left side) and experimental PXRD for complex **1** before and after solvent exchange with EtOH (right side).

Thermal evolution

Thermal evolution of as synthesised compound **1** shows that the material keeps its initial phase crystallinity up to 110 ºC. Above this temperature, new peaks appear in the diffractogram indicating the transformation into a different crystalline phase; this second phase, maintains its crystallinity up to 210 ºC and it must be noted that is very similar to the phase obtained when material was soaked in H_2O. Finally, compound **1** collapses and evolves into metallic residue CuO, obtained around 800 ºC.

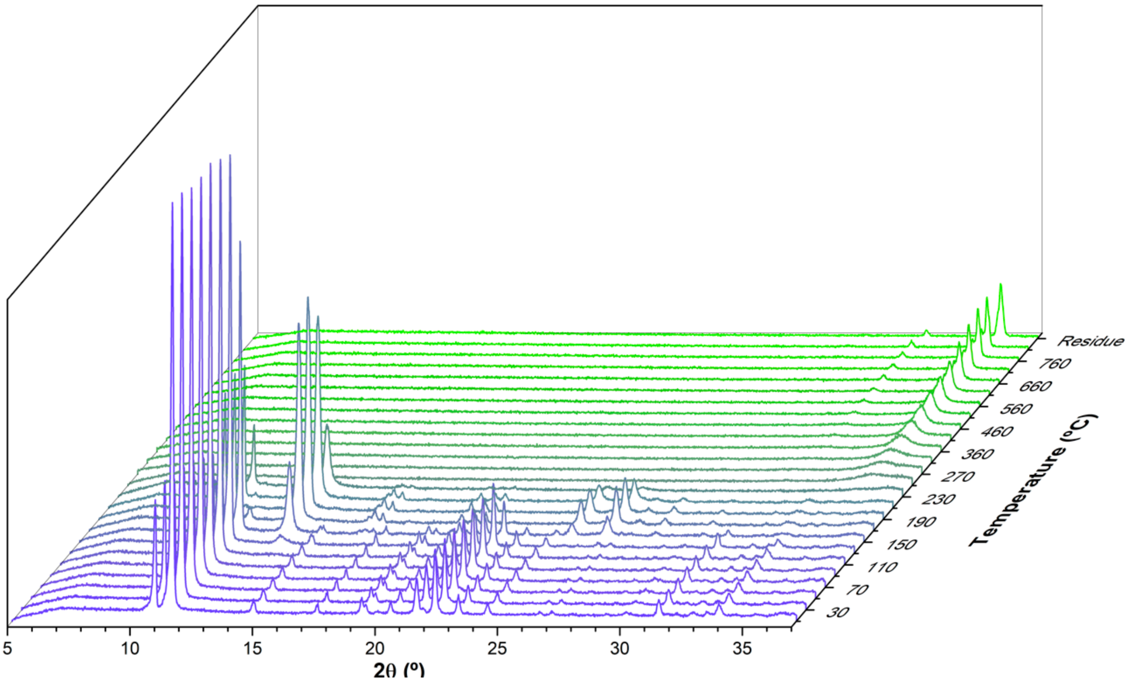

Figure S4. Thermal evolution of as synthesised compound **1.**

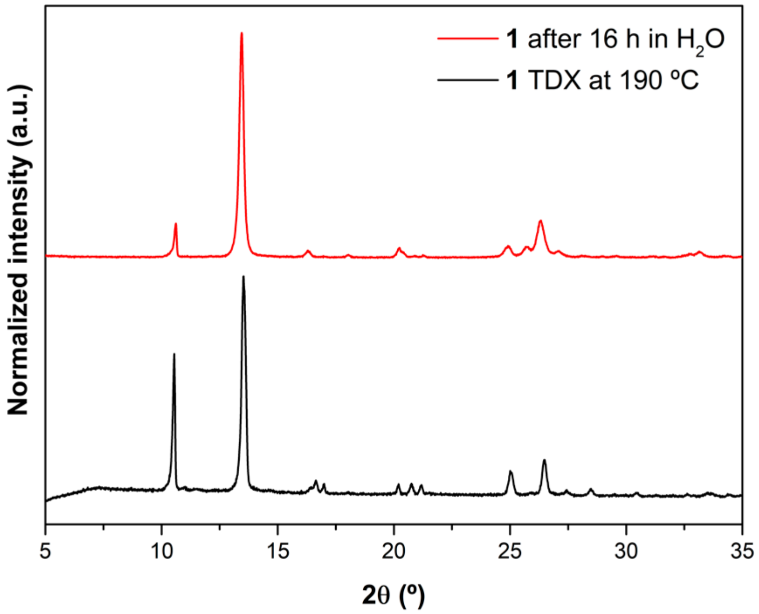

Figure S5. Comparison between the PXRD transformed phase of compound **1** obtained from thermal evolution at 190 ºC and the phase obtained soaking compound **1** in H_2O during 16 h.

MOF processing: Transformation into pellets and membranes

In order to incorporate MOFs into devices, bulk material shaping is of great importance [10–14], in this line, with the aim of exploring processing techniques compound **1** was transformed into pellets and membranes [12,13]. A home-made extrusion apparatus (composed by a syringe) allowed pellets to be compressed as binding agent, water was used. For pellets preparation, 100 mg of compound **1** were weighted and 100 µL was added to form a malleable paste. Subsequently, the paste was transferred into a syringe and compressed. With the aid of an external heating source, biding agent elimination allowed obtaining final pelletized material. With the aim of enhancing resistance, some of pellets were coated with polymer, in this case, polysulphone was used for that purpose. For polymer coating, pellet was submerged during 2 s in an already prepared polymer solution of CH_2Cl_2 containing 300 mg of polysulphone. Once dichloromethane being evaporated the coating dried and pellet got its final resistance.

Membrane preparation was carried out according the following procedure: 300 mg of polysylphone (PSF) were weighted and dissolved in 5 mL of CH_2Cl_2 to this dense solution, 100 mg of compound **1** were added and left stirring for 30 min. The remaining viscous solution was then cast in a glass petri dish and left unstirred at ambient conditions until complete evaporation of the solvents.

Once, compound **1** being transformed into pellets and membranes, the stability of these final processed materials was tested against moisture and temperature cycles. For that purpose, materials were placed for 72 h in a desiccator containing K_2SO_4 saturated solution which simulated 98% relative humidity (RH) ambient [15]. Afterwards, materials were characterised by PXRD.

Moisture stability

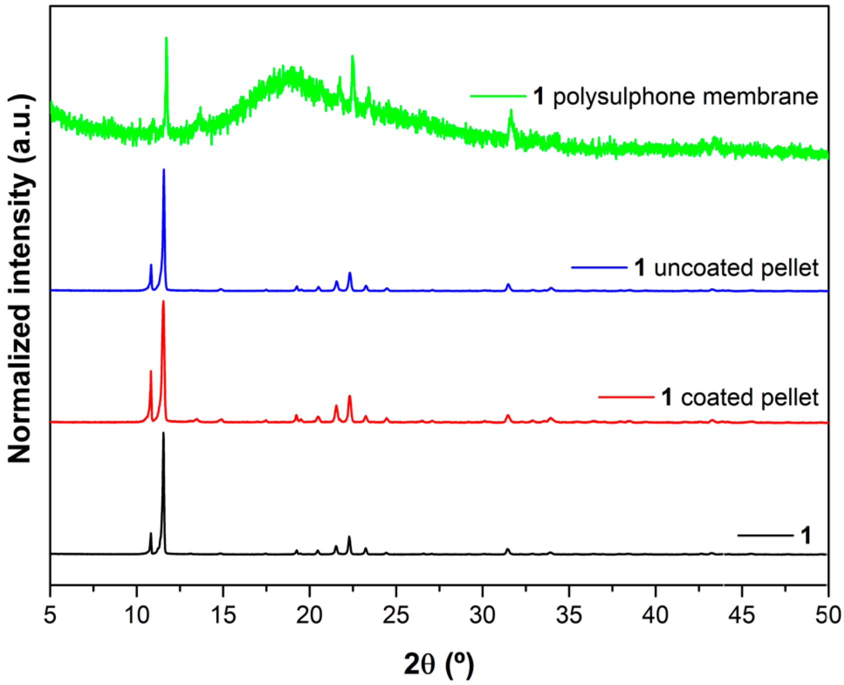

Figure S6. Powder X-ray diffractograms of the studied materials, as-synthesized and after processing into pellets and membranes subsequent moisture treatment.

PXRD results exhibit that compound **1** is stable and keeps its structure after being transformed into pellets or membranes and being exposed for 72 h at 98 % RH. Contrary to what happens when material is directly put in contact with water, that evolves into another crystalline phase, long exposure to humidity do not provoke material transformation and remains stable. Due to the amorphous nature of the polymer, only the main intense peaks of compound **1** stands over the amorphous background in polysulphone based membrane's PXRD.

Temperature stability

Once carrying out humidity tests, pellets were subsequently treated to temperature cycles. For that purpose, four heating and cooling down cycles were performed and materials were characterised by PXRD analysis. Therefore, uncoated pellets and polysulphone with coated were first tested against 98 % relative humidity (RH) for 72 h and later tested their thermal stability. For this purpose, temperature cycles of heating to 125 ^{o}C and cooling down to room temperature were carried out. After each cycle, a photograph of both pellets (coated and uncoated) was taken in order to check their integrity. Subsequently, PXRD analysis was performed after the second and forth cycle in coated and uncoated pellets.

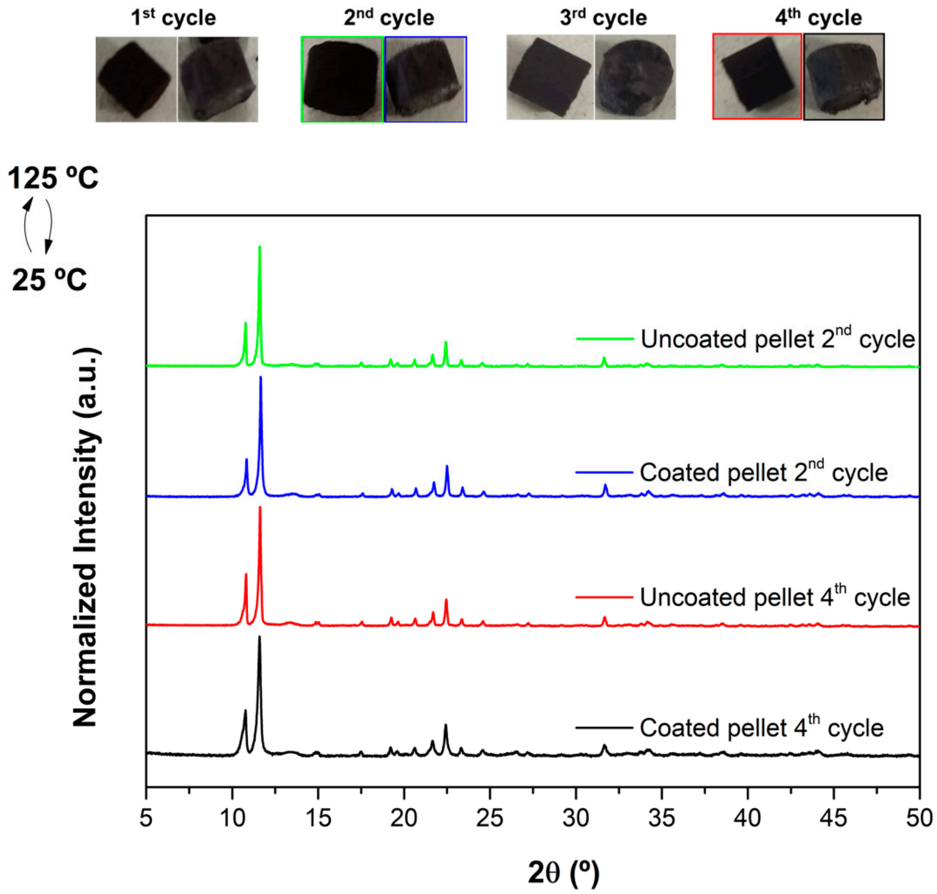

Figure S7. Powder X-ray diffractograms of the studied materials, as-synthesized and after processing into pellets after temperature-cycles.

Overall, the compound **1** coated and uncoated pellets resist perfectly to humidity and subsequent temperature cycles not only keeping their integrity but also their structure according to results obtained by PXRD. However, it must be noticed that coated pellets keep better their integrity because of the resistance apported by the polymer.

Water adsorption analysis

The water vapour adsorption isotherms were collected in a Dynamic Vapor Sorption apparatus from Surface Measurements Systems, using N_2 as the carrier gas (Air Liquide Alphagaz, less than 3 ppm H_2O, total flow of 200 sccm). Dry aliquots (16 mg) were loaded in a steel pan and suspended in the measuring chamber. The experiment started with a 2 h pre-treatment at 125 ºC, to completely dry the sample, followed by the isotherm at 25 ºC with increasing and decreasing RH steps from 0 to 98 %. Each humidity step was kept until the rate of change of mass per fixed time (dm/dt) was lower than 0.002 %, for a period of at least 10 min.

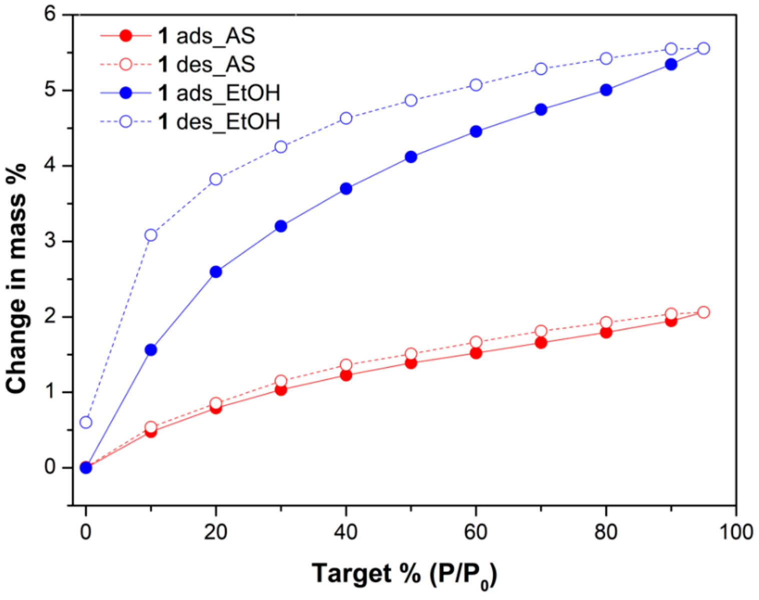

Figure S8. Water adsorption isotherm of compound **1** as synthesised (AS) and after solvent-exchange with EtOH for 16 h.

Adsorption properties

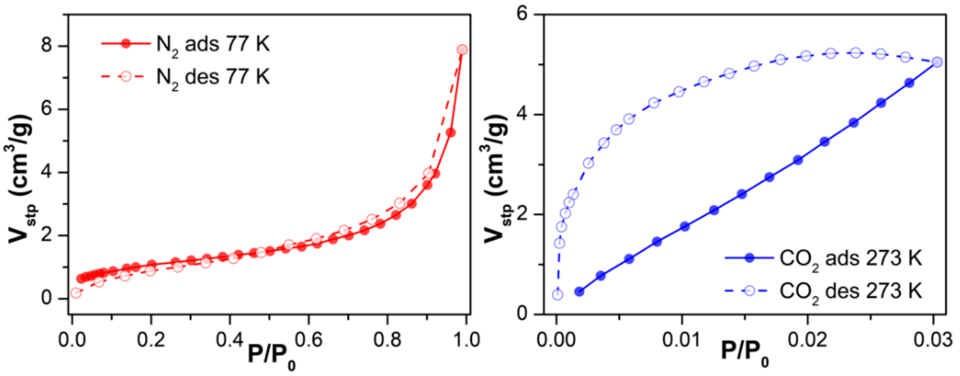

Figure S9. N_2 and CO_2 adsorption at desorption isotherms at 77 K and 273 K of **1**, upon outgassing at 170 ºC for 6 h.

FT-IR spectroscopy

Compound **1** exhibit a set of sharp and of medium intensity bands that corresponds to aromatic ring's C–H bond vibrations of the ligand are visible between 3073 cm^{-1} and 2989–2864 cm^{-1}. The following intense vibrations located in 1659–1426 cm^{-1} region are attributed to both the asymmetric stretching vibrations of the carboxylate groups and the aromatic C–C bonds. Moving to lower range of 1389–1252 cm^{-1}, symmetric stretching vibrations of the carboxylate groups appear in the spectrum. The remaining bands that are found at lower frequency can be attributed to the distortions originated in the aromatic ring and the carboxylate groups of the ligands.

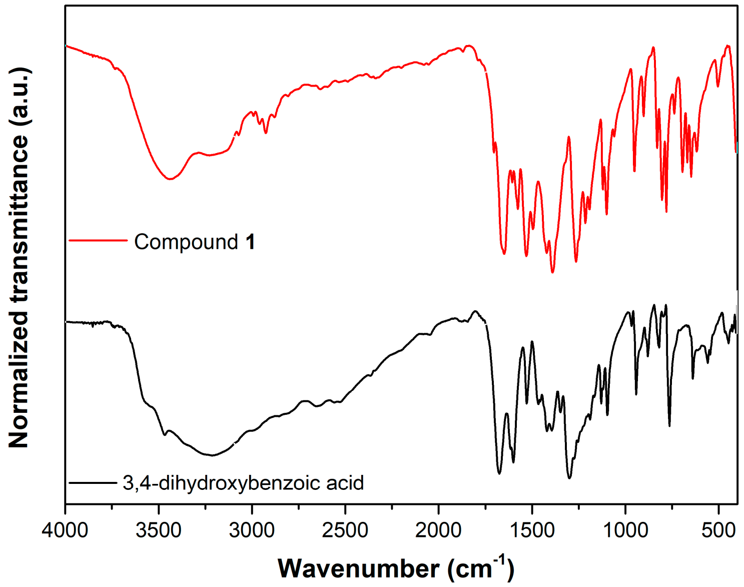

Figure S10. Infrared spectra of the ligand and compound **1**.

Scanning Electron Microscopy

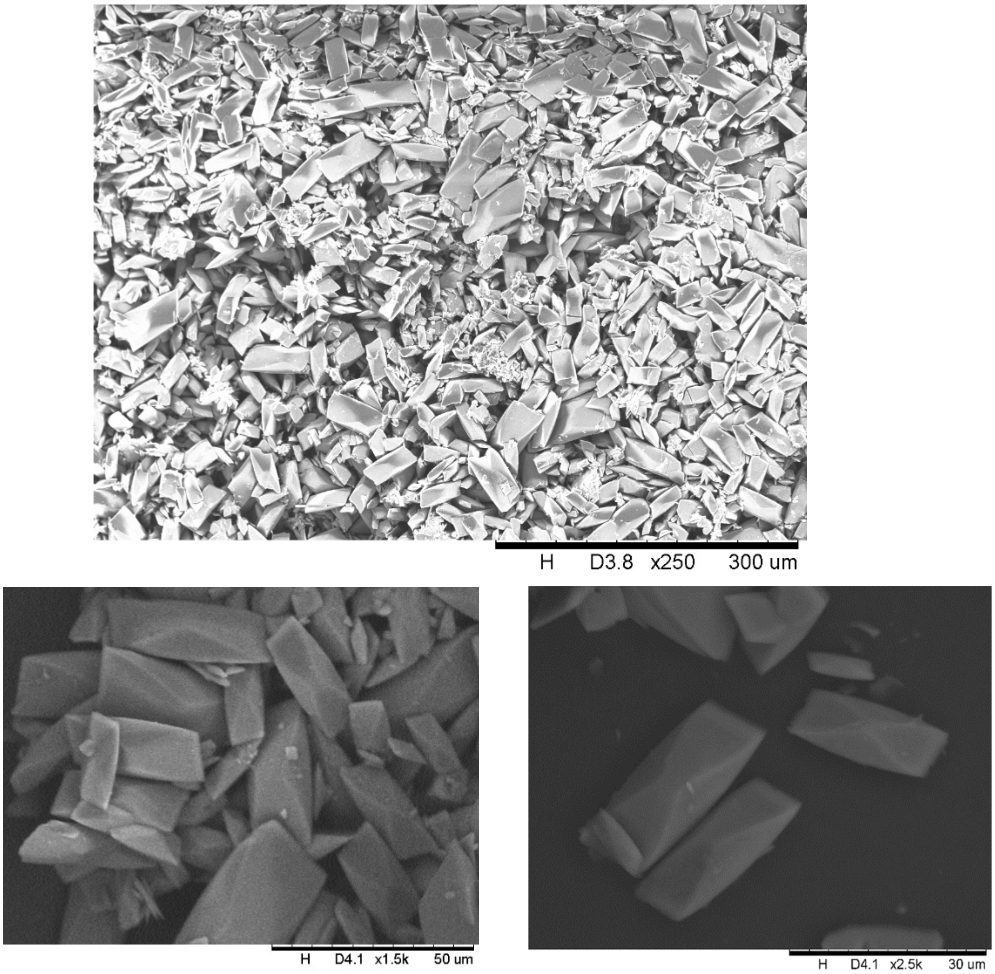

Figure S11. SEM images of compound **1**.

Powder X-ray diffraction analysis

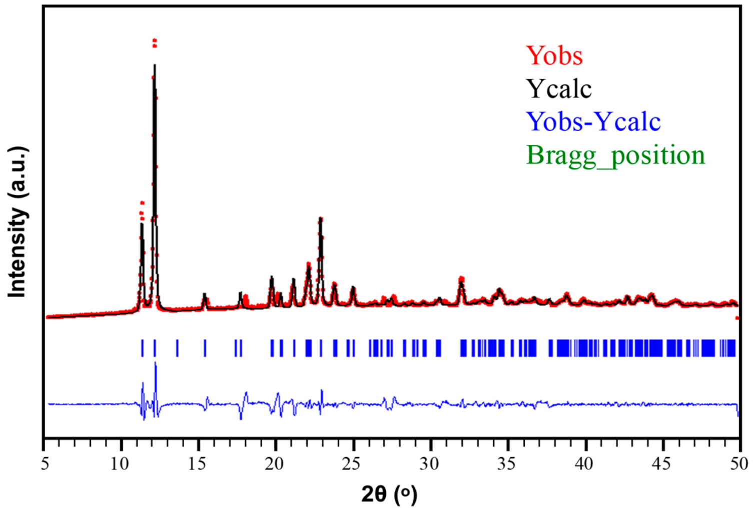

Figure S12. Figure of the pattern matching analysis and experimental PXRD for complex **1**.

Solvent stability

Compound **1** stability was examined in various solvents. For that purpose, 100 mg of MOF were suspended in a 4 mL of solvent and left agitating for 16 h. Afterwards, material was filtered off and left drying before carrying on powder X-ray diffraction (PXRD) analysis.

According to the PXRD, compound **1** is stable in EtOH after 16 h keeping its structure, contrary to what it happens in MeOH and H_2O. In the latter case, compound **1** completely evolves into a different crystalline product. However, in the former case, in MeOH, PXRD reveals that even if compound **1** most important peaks are present in the diffractogram, several new peaks appear. The position of those new peaks come in accordance with the evolved crystalline product obtained after soaking compound **1** for 16 h in water.

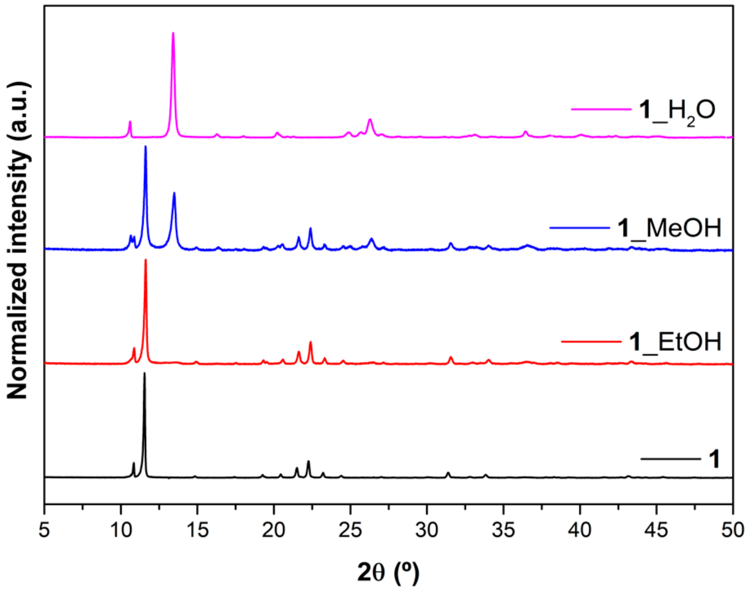

Figure S13. Experimental PXRD for complexes

Selected bond lengths and angles data

Single Crystal X-ray Diffraction

Table S4. Selected bond lengths (in Å) and bond angles (in º) for the Cu^{2+} coordination environments present in $[Cu_3L_2(DMF)_2]$ (**1**)

Cu1—O4^{i}	1.924 (3)	Cu2—O3	1.927 (3)
Cu1—O2	1.943 (2)	Cu2—O3^{iii}	1.927 (3)
Cu1—O1	1.948 (2)	Cu2—O1^{iv}	1.960 (2)
Cu1—O2^{ii}	1.953 (2)	Cu2—O1^{v}	1.960 (2)
Cu1—O8	2.296 (3)	Cu2—O8^{iv}	2.591 (3)
O4^{i}—Cu1—O2	169.30 (12)	O3—Cu2—O3^{iii}	180.0
O4^{i}—Cu1—O1	95.99 (11)	O3—Cu2—O1^{iv}	88.65 (11)
O2—Cu1—O1	84.46 (10)	O3^{iii}—Cu2—O1^{iv}	91.35 (11)
O4^{i}—Cu1—O2^{ii}	99.17 (11)	O3—Cu2—O1^{v}	91.35 (11)
O2—Cu1—O2^{ii}	78.58 (11)	O3^{iii}—Cu2—O1^{v}	88.65 (11)
O1—Cu1—O2^{ii}	161.18 (10)	O1^{iv}—Cu2—O1^{v}	180.00 (14)
O4^{i}—Cu1—O8	94.65 (13)	O3—Cu2—O8^{iv}	92.68 (12)
O2—Cu1—O8	96.04 (12)	O3^{iii}—Cu2—O8^{iv}	87.32 (12)
O1—Cu1—O8	89.57 (12)	O1^{iv}—Cu2—O8^{iv}	81.20 (10)
O2^{ii}—Cu1—O8	100.21 (12)	O1^{v}—Cu2—O8^{iv}	98.80 (10)

Symmetry codes: (i) *x*−1/2, −*y*+3/2, *z*−1/2; (ii) −*x*+1, *y*, −*z*+1/2; (iii) −*x*+2, −*y*+2, −*z*+1; (iv) *x*+1/2, −*y*+3/2, *z*+1/2; (v) −*x*+3/2, *y*+1/2, −*z*+1/2.

1 after being soaked for 16 h in several solvents.

References

1. J. Rodríguez-Carvajal FULLPROF 2000,version 2.5d,. *FULLPROF 2000,version 2.5d, Lab. Léon Brillouin (CEA-CNRS), Cent. d'Études Saclay, Gif sur Yvette Cedex, Fr.* 2000.

2. Kottke, T.; Stalke, D. Crystal handling at low temperatures. *urn:issn:0021-8898* **1993**, *26*, 615–619, doi:10.1107/S0021889893002018.

3. SAINT+, Data Integration Engine v. 8.37a©, 1997-2015, Bruker AXS, Madison, Wisconsin, USA.

4. Krause, L.; Herbst-Irmer, R.; Sheldrick, G.M.; Stalke, D. Comparison of silver and molybdenum microfocus X-ray sources for single-crystal structure determination. *J. Appl. Crystallogr.* **2015**, *48*, 3–10, doi:10.1107/S1600576714022985.

5. Sheldrick, G.M. SADABS User Manual. *SADABS; Univ. Göttingen Göttingen, Ger.* **2008**.

6. Sheldrick, G.M. SHELXT - Integrated space-group and crystal-structure determination. *Acta Crystallogr. Sect. A Found. Crystallogr.* **2015**, *71*, 3–8, doi:10.1107/S2053273314026370.

7. Hübschle, C.B.; Sheldrick, G.M.; Dittrich, B. ShelXle: a Qt graphical user interface for SHELXL. *J. Appl. Cryst* **2011**, *44*, 1281–1284, doi:10.1107/S0021889811043202.

8. K. Brandenburg, DIAMOND, Version 3.2f. Crystal Impact GbR, Bonn, Germany, 1997-2010.

9. Llunell, M.; Casanova, D.; Cirera, J.; Bofill, J. M.; Alemany, P.; Alvarez, S.; Pinsky, M.; Avnir, D. Program for the Stereochemical Analysis of Molecular Fragments by Means of Continuous Shape Measures and Associated Tools. *SHAPE, v1.1b; Barcelona, Spain* **2005**, 1–35.

10. Ren, J.; Langmi, H.W.; North, B.C.; Mathe, M. Review on processing of metal-organic framework (MOF) materials towards system integration for hydrogen storage. *Int. J. Energy Res.* **2015**, *39*, 607–620, doi:10.1002/er.3255.

11. Liu, X.M.; Xie, L.H.; Wu, Y. Recent advances in the shaping of metal–organic frameworks. *Inorg. Chem. Front.* **2020**, *7*, 2840–2866, doi:10.1039/C9QI01564G.

12. Ntouros, V.; Kousis, I.; Pisello, A.L.; Assimakopoulos, M.N. Binding Materials for MOF Monolith Shaping Processes: A Review towards Real Life Application. *Energies* **2022**, *15*, doi:10.3390/en15041489.

13. Figueira, F.; Mendes, R.F.; Domingues, E.M.; Barbosa, P.; Figueiredo, F.; Paz, F.A.A.; Rocha, J. Easy processing of metal-organic frameworks into pellets and membranes. *Appl. Sci.* **2020**, *10*, doi:10.3390/app10030798.

14. Ma, Q.; Zhang, T.; Wang, B. Shaping of metal-organic frameworks, a critical step toward industrial applications. *Matter* **2022**, *5*, 1070–1091, doi:10.1016/J.MATT.2022.02.014.

15. F E M O'Brien The Control of Humidity by Saturated Salt Solutions. *J. Sci. Instrum.* **1948**, *25*, 73–76.
